# Anticancer properties of nimbolide and pharmacokinetic considerations to accelerate its development

**DOI:** 10.18632/oncotarget.8316

**Published:** 2016-03-24

**Authors:** Lingzhi Wang, Do Dang Khoa Phan, Jingwen Zhang, Pei-Shi Ong, Win Lwin Thuya, Ross Soo, Andrea Li-Ann Wong, Wei Peng Yong, Soo Chin Lee, Paul Chi-Lui Ho, Gautam Sethi, Boon Cher Goh

**Affiliations:** ^1^ Cancer Science Institute of Singapore, National University of Singapore, Singapore; ^2^ Department of Pharmacology, National University Health System, Singapore; ^3^ Department of Pharmacy, National University of Singapore, Singapore; ^4^ Department of Haematology-Oncology, National University Health System, Singapore

**Keywords:** nimbolide, anticancer property, pharmacodynamics, pharmacokinetics, toxicology

## Abstract

Nimbolide is one of the main components in the leaf extract of *Azadirachta indica* (*A. indica*). Accumulating evidence from various *in vitro* and *in vivo* studies indicates that nimbolide possesses potent anticancer activity against several types of cancer and also shows potential chemopreventive activity in animal models. The main mechanisms of action of nimbolide include anti-proliferation, induction of apoptosis, inhibition of metastasis and angiogenesis, and modulation of carcinogen-metabolizing enzymes. Although multiple pharmacodynamic (PD) studies have been carried out, nimbolide is still at the infant stage in the drug development pipeline due to the lack of systematic pharmacokinetic (PK) studies and long-term toxicological studies. Preclinical PK and toxicological studies are vital in determining the dosage range to support the safety of nimbolide for first-in-human clinical trials. In this review, we will provide a comprehensive summary for the current status of nimbolide as an anticancer and chemopreventive lead compound, and highlight the importance of systematic preclinical PK and toxicological studies in accelerating the process of application of nimbolide as a therapeutic agent against various malignancies.

## INTRODUCTION

**Figure 1:**
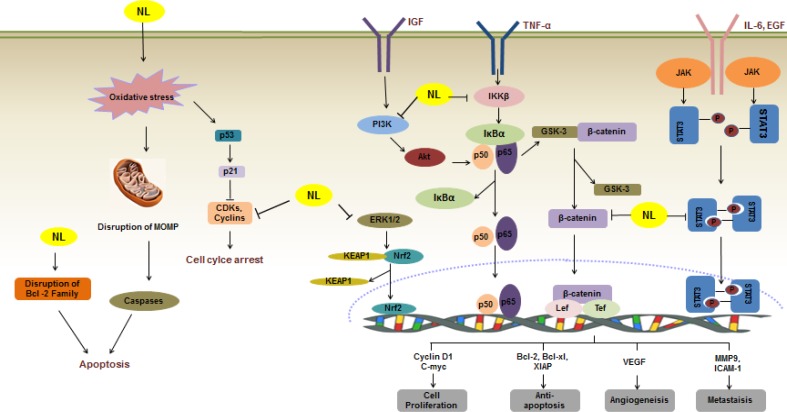
A schematic illustration of the molecular mechanisms of the anticancer action of nimbolide [91] Nimbolide induces apoptosis through diverse molecular mechanism(s). Nimbolide disrupts MOMP, which promotes caspases activation leading to apoptosis. Nimbolide also reduces the level of CDKs and cyclins, causing cell cycle arrest. Nimbolide also abrogates various signaling cascades including MAPK (ERK1/2), JAK2/STAT3 and PI3K/Akt, leading to suppression of proliferation of a wide variety of human cancer cells. Nimbolide disrupts the Nrf2-KEAP1 complex and promotes the release of Nrf2, thus increasing levels of antioxidant and detoxification enzymes. Nimbolide suppresses IκBα degradation and prevents nuclear translocation of p65, therefore repressing the expression of various genes involved in cell proliferation, anti-apoptosis, angiogenesis and metastasis. Inhibition of the NF-κB pathway also reduces the dissociation of GSK-3β from β-catenin, hence restraining the Wnt/β-catenin signaling pathway. →: activate/induce; Long Left Tack: suppress/inhibit.

*Azadirachtaindica*, commonly known as neem, is a tree belonging to the Meliceae family, which is native to India and the Indian Subcontinent. Today, neem is well distributed across the world in at least 30 countries including Asia, Africa, and America [1]. Natural compounds that have been identified from various parts of *A.indica* include azadirachtin, salannin, nimbolide, nimbin, and nimbic acid [2]. Among them, nimbolide is a major tertranortriterpenoid, isolated from the leaves of *A.indica*. Hence, nimbolideis accessible and affordable, owing to the abundance of neem trees in tropical regions. More importantly, nimbolide is well known for its many uses, such as anti-malaria [3], antibacterial activity against *S.aureus* and *S.coagulase* [4], anti-feedant [5], and insecticidal activity [6]. In addition, nimbolidehas been found to possess antioxidant effect and free radical scavenging activities. In comparison toazadirachtin and ascorbic acid (vitamin C), nimbolide was shown to be a more potent antioxidant [7]. Furthermore, nimbolidehas been identified as one of the active ingredients of neem extract, a widely available herbal product used in traditional Indian Ayurvedic medicine to treat acne, wound, gastric ulcer, and infections [8, 9]. In a study by Cohen and co-workers, nimbolide was reported to be the most potent cytotoxic substance among 6 limonoids examined [10]. Through investigation of nimblolide's action in multiple cancer cell lines, various molecular targets were thus identified. Hence, the mechanistic interpretation of its pharmacodynamic actions can be derived. The major signaling pathways by which nimbolide exerts its effects are represented in Figure 1.

In addition, the anticancer and cancer preventive effects of nimbolide were observed in several animal studies [11–13], thereby providing strong evidence to support its further development as a therapeutic agent against cancer. In accordance with the drug development pipeline [14], nimbolide is now at the stage of preclinical pharmacological investigation, where pharmacodynamics (PD), toxicology, and pharmacokinetic (PK) studies should be performed before the drug can proceed to the clinical testing phase. Although the development of various separation techniques [15–18] has facilitated a highly efficient extraction of nimbolide for use in many *in vitro* experiments to elucidate its potential therapeutic actions, the absence of information on its absorption, distribution, metabolism, and elimination (ADME), as well as its toxicity, in turn hampers further clinical studies on this natural compound. Therefore, research on its PK characteristics and toxicological profile should be carried out to facilitate and accelerate its development as an anticancer drug candidate for clinical trials. The objectives of the present review are to summarize the current status of nimbolideas a chemopreventive and chemotherapeutic agent in specific cancer types based on pre-clinical studies, as well as to highlight the importance of performing toxicological and PK studies that will lay the foundation for its early-phase clinical investigation.

## METHODOLOGY

Web of Science and Google scholar database searches were conducted during August -September 2015 to identify and retrieve articles related to nimbolide and its derivatives. Search terms included “nimbolide”, ”neem”, “Azadirachtaindica “and” nimbolide derivatives”. The Boolean operator ‘OR’ was placed between each of the search words. The reference list of each article was analysed in detail to identify additional relevant articles. The reviewer read each article in full text (n=70), evaluated its relevance and recorded the findings in a table. The table included a summary of each article's content and its relevance on a scale rating (not relevant, relevant, very relevant). The “relevant” and “very relevant” articles (n=55) were further reviewed to identify key pharmacodynamic properties of nimbolide.

## CHEMICAL STRUCTURE AND PROPERTIES OF NIMBOLIDE

**Figure 2:**
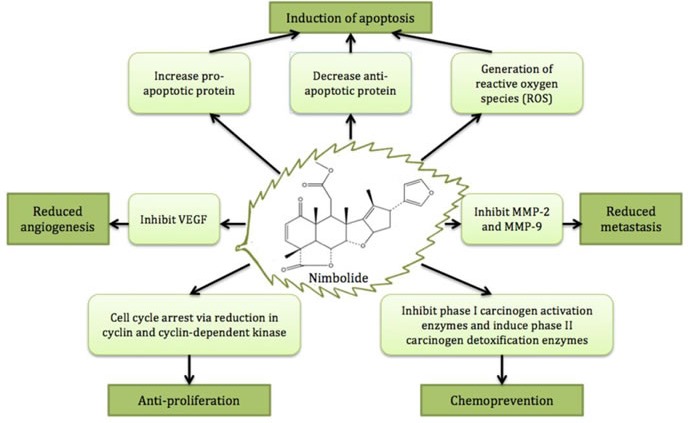
The major anticancer activities and cancer preventive effect of nimbolide

The molecular formula of nimbolide [systematic name: (4alpha, 5alpha, 6alpha, 7alpha, 15beta, 17alpha)-7,1521, 23-diepoxy-6-hydroxy-4,8-dimethyl-1-oxo-18,24-dinor-11,12-secochola-2,13,20,22-tetraene-4,11-dicarboxylic acid gamma-lactone methyl ester], is C_27_H_30_O_7_ [19]. Nimbolide has a decalinskeleton [20] and belongs to a group of tetranortriterpenoids called C-secoMeliacins [2]. The 2D chemical structure of nimbolide is illustrated in Figure 2.

Nimbolide was first extracted from fresh leaves of a Nigerian sample of *A.indica*by using petroleum spirit. Nimbolide has molecular mass 466, melting point 245-247°C and optical rotation of [a]_D_+2060. Its infrared radiation (IR) spectrum shows carbonyl absorptions at 1665 (cyclohexenone), 1720 (CO_2_CH_3_) and 1770cm^−1^ (γ-lactone) [17]. Using this method, nimbolide was obtained as colorless plates following crystallization from acetone [15]. Another method of nimbolide isolation by column chromatography from crude extract of *A.indica*h as also been reported [16].

The significant cell cytotoxicity and anticancer effect of nimbolide was most likely due to the presence of anα, β-unsaturated ketone structural element. In addition, a γ-lactone moiety was also reported to be responsible for its cytotoxicity [15]. Furthermore, structural modifications of nimbolide to various amide derivatives have conferred improved cytotoxicity. In 2006, Sastry et al. reported that two of the nimbolide derivatives possessed stronger inhibitory activities than nimbolide against six cancer cell lines, namely, HT-29 (colon), SW-620 (colon), HOP-62 (lung), A-549 (lung), PC-3 (prostate), and OVCAR-5 (ovary) [21].

## ANTI-CANCER ACTIVITIES

Various PD studies of nimbolide on different cancer types have been conducted. There is strong evidence to support its activity against a wide range of malignancies from *in vitro* studies and *in vivo* animal models.

### *In vitro* studies

Nimbolide has been shown to exhibit anticancer effects on a variety of cancer cell lines through disturbing the cell cycle of cancer cells. For instance, in human colon cancer cells, nimbolide induced cell cycle arrest and abrogated cell growth [22]. Nimbolide also exerted a strong inhibitory effect on the growth of colon cancer (HT-29) cellsdue to cell cycle arrest. In another study [23], flowcytometric analysis of U937 cells (leukemic cell lines) showed that nimbolide treatment (1–2.5 μM) resulted in cell cycle disruption by decreasing the number of cells in G0/G1 phase. Besides these, the anti-proliferation effect on cancer cells was also evident by the reduction of Ki-67 protein [24] in Waldenstrom macroglobulinemia (a type of B lymphocyte cancer) [11] and glioblastoma multiforme [12].

More importantly, nimbolide showed a strong apoptotic effect on cancer cells. It also has an inhibitory role on metastasis and angiogenesis of cancer cells. For example, nimbolide treatment resulted in 50% inhibition of the prostate cancer PC-3 cell line at a concentration of 2μM. This was accompanied by the induction of cellular apoptosis [25]. Similarly, nimbolide was shown to induce apoptosis in human osteocarcinoma [26], Waldenstrom macroglobulinemia (WM) [11], human cervical cancer [27], and hepatocarcinoma cells [28]. Additionally, an *in vitro* study further indicated that nimbolide was able to combat metastasis and angiogenesis of cancer cells [29]. For instance, nimbolide have been shown to effectively decrease breast cancer cell invasion and migration via the transwell invasion and wound healing assays [30].

In order to comprehensively analyze the anticancer activity of nimbolide, the half inhibitory concentrations (IC_50_) of nimbolide in different cancer types were compared, ranked, and presented in Table 1. The IC_50_values for various cancer types tested range between 0.2 to 15 μM. WM emerged as the best target for nimbolide with the lowest IC_50_value, which was in the nanomolar range. Since nimbolide showed remarkable potency against WM, the development and research of nimbolide as potential drug candidate for WM should be prioritized. In addition, nimbolide has also been found to be quite effective against leukemia, choriocarcinoma and colon cancer, with IC_50_values slightly above 1 μM [22, 23, 31]. Further investigation on these 3 cancer types is also warranted.

**Table:** Summary of *in vitro* IC_50_ of nimbolide in different cancer types

Rank	Cancer Type	*In Vitro* Concentration (IC_50_)	Reference
1	Waldenstrom Macroglobulinemia: cancer of B lymphocytes	0.20 μM	[11]
2	Leukemia	1.12 μM	[23]
3	Choriocarcinoma: trophoblastic cancer	1.19 μM	[31]
4	Colon cancer	1.25 μM	[22]
5	Melanoma: skin cancer	1.74 μM	[23]
6	Prostate cancer	2.00 μM	[25]
7	Glioblastoma multiforme: primary brain tumour	3.00 μM	[12]
8	Breast Cancer	4.00 μM	[92]
9	Osteocarcinoma: bone cancer	4.30 μM	[10]
10	Cervical cancer	5.00 μM	[27]
10	Liver cancer	5.00 μM	[28]
12	Neuroblastoma	5.20 μM	[10]
13	Lung cancer	15.6 μM	[21]

### *In vivo* studies

Beside *in vitro* investigations, *in vivo* studies have been performed on the preclinical effect of nimbolide against colorectal, lymphoma, andbrain cancers. Firstly, nimbolide was shown to possess a dose-dependent anticancer effect on colorectal cancer. When nimbolide was injected intraperitoneally (*i.p.*) for 10 days at a dose of 5mg/kg, a 67% reduction in the volume of colorectal cancer xenografts in mice was observed. At a higher dose of 20 mg/kg, an even more substantial and remarkable 90% reduction in tumour volume was achieved [13]. This was further accompanied by inhibition of angiogenesis and tumour metastasis, there by highlighting the promising activity of nimbolide against this cancer.

However, incongruency between the anticancer potency of nimbolide between *in vivo* doses and *in vitro* IC_50_ has been reported. In a WM tumour xenografted mouse study [11], Chitta et al. reported that nimbolide was only effective against WM tumours at very high doses of 100-200 mg/kg, *i.p.* after 26days of treatment. These doses arehowever very close to the previously reported median lethal dose (LD_50_) of nimbolide(280 mg/kg) in adult female mice [32]. Hence, we can conclude that nimbolide has very weak anticancer activity against WM tumours *in vivo* despite its high sensitivity (IC_50_ = 0.2 μM) against WM cells *in vitro*. In contrast to the high doses used in WM study, significantly lower doses (5-20 mg/kg) were administered via the same *i.p.*route in a colorectal cancer study. Here, a good anticancer effect was achieved although nimbolide was less potent in colorectal cancer cells (IC_50_ = 1.25 μM). Likewise, in another study on glioblastoma multiforme, nimbolide was very potent on brain tumour *in vivo* at a dose of 0.01 mg/kg via intravenous (*i.v.*) injection for 7 days, while glioblastoma cells has been found to be much less sensitive to nimbolide, with a relatively high *in vitro* IC_50_ (3 μM) [12]. Hence, systematic PK studies need to be carried out to establish the relationship between the *in vivo* doses and the maximum achievable concentrations in blood and tumour site, which can help to elucidate the gap between the *in vitro* and *in vivo findings*.

## CHEMOPREVENTIVE EFFECT

In addition to its anticancer effect, nimbolide showed remarkable chemopreventive property. A chemopreventive agent perturbs various steps in cancer initiation, promotion, and progression. For instance, chemopreventive agents hinder tumour initiation by preventing interaction between carcinogens or reactive free radicals and DNA, hence lowering the chance of DNA damage and mutations [33]. Nimbolide acts as an effective chemopreventive agent since it modulates the biotransformation of carcinogens. Carcinogens upon entering the body are either activated or deactivated by xenobiotic-metabolizing enzymes [34–36], which will be elaborated in the next section. The chemo preventive effects of nimbolide were reported in an *in vivo* study using 7,12-dimethylbenz[a]anthracene (DMBA)-induced hamster buccal pouch (HBP) carcino genesis as a model [7]. In this study, upon oral administration of 0.01 mg/kg of nimbolide for 14 weeks, nimbolide reduced the incidence of tumour and pre-neoplastic lesions, there by highlighting its potent chemopreventive activity and potential as a candidate for cancer prevention.

## MECHANISM OF ACTIONS

The major anticancer activities and cancer preventive effect of nimbolideare depicted in Figure 2.

### Inhibition of cell growth and proliferation

Cell growth and proliferation are mediated by progression through cell cycle, which is tightly regulated by a group of proteins called cyclin-dependent kinases (CDKs). CDKs bind to a regulatory protein called cyclin to form an activated complex, which drives the transition of cells from one phase to another [37].

Nimbolide caused cell cycle arrest at G1/S phase. Evidently, nimbolide was found to reduce cyclin A level, which is required for colon cancer cells to proceed through S phase, hence inducing cell cycle arrest and resulting ininhibition of cell growth [22]. Similarly, nimbolide also reduced the expression of CDK2/cyclin E complex and increased the level of cyclin D2, thereby preventing colon cancer cells from entering the S phase of the cell cycle [37–39]. In addition, nimbolide directly inhibited CDK4/CDK6 kinase activity, leading to hypophosphorylation of the retinoblastoma protein (Rb), which in turn downregulated the expression of cyclin E, resulting in cell cycle arrest at G1/S and suppressionof glioblastoma growth [12].

Apart from G1/S phasearrest, nimbolidealso caused cell cycle arrest at both the G0/G1 and G2/M phases. For instance, nimbolide significantly suppressed the viability of HeLa cells by inducing cell cycle arrest at G0/G1 phase accompanied by p53-dependent p21 accumulation and downregulation of cyclin B as well as cyclin D1 [27]. In nimbolide-treated HT-29 colorectal cancer cells, a substantial downregulationG2/M cell cycle checkpoint proteins, CHK2 and Rad17 were noted [38]. Taken together, nimbolide exhibited inhibitory activity on several CDK/cyclin molecules, there by resulting in cell cycle arrest.

### Induction of apoptosis

Nimbolide can induce apoptosis in cancer cells by modulation of apoptotic proteins via both intrinsic and extrinsic pathways of apoptosis [40]. The intrinsic pathway is a mitochondria-mediated pathway while the extrinsic pathway is mediated by death receptors (DR).

In the intrinsic pathway, the death signal triggers mitochondria to activate Bax, which forms holes in the outer membrane, facilitating the release of cytochrome c [41]. Cytochrome c interacts with Apaf-1 to form the apoptosome, which activates initiator caspase-9. Caspase-9 in turn activates effector caspase-3, which causes fragmentation of the nucleus, disruption of the cytoskeleton, membrane blebbing, and cellfragmentation [42]. Antiapoptotic proteins and proapoptotic proteins regulate the level of activation of caspase-3. Nimbolide treatmentdecreased the expression of antiapoptotic proteins(Bcl-xL, Bcl-2, survivin, caspase inhibitor molecules) and increased the expression of proapoptotic proteins (cytochrome c, Bax, Bad, Bid, cleaved caspases) in prostate cancer cells [25].

Moreover, a variety of signaling pathways regulate apoptosis by controlling the expression or activity of proapoptotic members of the Bcl-2 family [43]. Activation of the tumor suppressor p53, for example, can induce programmed cell death. Cytosolic p53 can in turn inhibit anti-apoptotic Bcl-xL and promote cytochrome c release. Using this mechanism, nimbolide acted by up-regulation of p53 levelin HeLa cells, thereby priming these cells towards apoptosis by destabilizing the mitochondria [44, 45].

In the extrinsic pathway, extracellular death signals such as death ligand, Fas ligand, tumour necrosis factor (TNF), and TNF-related apoptosis-inducing ligand(TRAIL) bind to death receptors (DRs), which activates caspase-8 [46–48]. Caspase-8 activates caspase-3, leading to apoptosis. Nimbolide up-regulated the expression of both DR5 and DR4 in chronic myeloid leukemia (KBM-5), multiple myeloma (U266), embryonic kidney carcinoma (A293), pancreatic adenocarcinoma (AsPC-1), and breast adenocarcinoma (MDA-MB-231) cells, resulting in activation of the extrinsic apoptotic pathway [49].

Moreover, for DRs up-regulation by nimbolide, activation of ERK and p38 MAPK were required. In nimbolide-treated colorectal cancer cells, apoptosis proceed by activation of caspase3/9 and ERK1/2 in the MAPK signaling pathway [49]. Furthermore, nimbolide resulted in generation of reactive oxygen species (ROS), which are required for ERK activation, which in turn up-regulated DRs and triggered apoptosis via the extrinsic pathway [27, 31]. ROS also induced lipid peroxidation of cellular membranes, generating toxic metabolites such as malondialdehyde (MDA) that can react with DNA to form adducts to induce apoptosis.

Nimbolidecan potentiate apoptosis induced by TNF-α in human leukemia cells [50]. Tumour necrosis factor (TNF-α) is a multifunctional inflammatory cytokine that also induces apoptosis of cancer cells via the extrinsic pathway [51]. Apart from its own antitumour effects, nimbolde can sensitize cancer cells to the effects of other anti-cancer agents such as TNF-related apoptosis-inducing ligand (TRAIL) [49]. In MCF-7 breast cancer cells, nimbolide and TRAIL were minimally effective in inducing apoptosis as single agents. However, when used in combination, significant apoptosis was observed in these cells, thereby extending the therapeutic efficacy of both agents. More importantly, this combination showed selectivity for cancer cells, as it was unable to evoke similar apoptosis in normal breast cells.

Another signaling cascade that nimbolide targets to decrease proliferation and enhance apoptosis of cancer cells is the Insulin-like Growth Factor I (IGF-1) pathway. IGF-I receptor is a receptor tyrosine kinase that can activate two different downstream effector cascades. Under the first scenario, Ras, Raf and mitogen-activated protein kinase (MAPKs) are activated, resulting in the transcription of genes that promote sustained proliferation [52]. In the second case, phosphoinositide 3-kinase (PI3K)/Aktis constitutively activated, which can regulate cellular survival and antiapoptotic signaling events [53]. Nimbolide was found to significantly inhibit IGF-1-mediated PI3K/Akt and MAPK signaling in human MCF-7 breast cancer cells, thereby inducing apoptosis and inhibiting proliferation of these cells [54].

Nimbolide can also act on a deregulated nuclear factor kappa-B(NF-κB) oncogenic pathway. NF-κB is a pro-inflammtory transcription factor involved in carcinogenesis, oncogenic progression, and apoptosis evasion [55]. NF-κB is inactivated in the cytoplasm by interacting with inhibitory Iκβ proteins such as IKKα and IKKβ [56]. This interaction results in the inactive NF-κB complex being primarily in the cytoplasm due to a strong nuclear export signal in Iκβ. In many cancer cells, NF-κB is constitutively active and located in the nucleus due to incessant stimulation of the IKK pathway that degrades Iκβ, while in some other cases, the Iκβ gene is mutated and dysfunctional [57–60]. As a result, NF-κB can enter the nucleus and promote the expression of multiple genes involved in tumor initiation, promotion and progression [56, 61]. Nimbolide was found to inhibit IκB degradation and prevent nuclear translocation of NF-κB. This subsequently caused cell cycle arrest by downregulating numerous genes involved in cellular proliferation [62]. Nimbolide can induce apoptosis through inactivation of NF-κB. This led to significant suppression of Bcl-2 with concomitant increase in the expression of Bax, cytochromec, and Smac/DIABLO [28]. Furthermore, nimbolide attenuated the NF-κB mediated Wnt/β-catenin signaling pathway in HepG2 cells. Wnt is a family of glycoproteins that regulates the amount of the transcriptional co-activator β-catenin that controls key developmental gene expression programs during embryonic development and tissue homeostasis. Hence, mutations in the Wnt pathway correlate to human birth defects and cancer [63]. Abrogation of NF-κB and Wnt signaling by nimbolide stimulatedcaspase-mediated apoptosis in HepG2 cells [28]. Unpublished data from our group also indicate that nimbolide can substantially abrogate the activation of another important oncogenic transcription factor, signal transducer and activator of transcription 3 (STAT3) in diverse prostate cancer cells. This effect was found to be mediated via an increased production of reactive oxygen species due to GSH/GSSG imbalance.

### Reduction in tumor metastasisandangiogenesis

Tumour metastasis accounts for approximately 90% of all cancer-related deaths [64]. The cancer cells from the primary site migrate to other organs via either the blood stream (haematogenous spread) or the lymphatic system (lymphatic spread), and form new tumours at the secondary sites [65]. The role of matrix metallo proteinases (MMP) and urokinase plasminogen activator (uPA) in matrix degradation and tumour invasion has been well studied [66–68]. MMP and uPA are regulated by tissue inhibitor of matrix metalloproteinases (TIMP) [69].

Nimbolide reducedphorbol 12-myristate 13-acetate(PMA)-induced tumor cell migration and invasion by inhibition of MMP via NF-κB [22]. The downregulation of proinvasive and angiogenic proteins, with up-regulationof their inhibitors by nimbolide that was observed in this study was in line with the anti-invasive and anti-angiogenic potential of neem preparations [70]. In addition, nimbolide reduced mRNA expression of MMP-2, MMP-9, uPA and uPA receptor in breast cancer cells (MCF-7) [30].

Chemokine (C-C motif) ligand(CCL) is a pro-inflammatory chemokine and associates with its receptor, C-C chemokine receptor type (CCR). CCL2 recruits inflammatory leukocytes (such as tumour-associated macrophages) in the tumour microenvironment and facilitates cancer cell progression [71]. CXC ligand (CXCL12) drives tumour invasion by induction of MMP-9 [72]. CXCR4 is responsible for metastatic implantation into other organs [73]. For instance, nimbolide treatment (2, 4 μM in breast cancer MCF-7 cells) was found to significantly reduce CCL2, CXCR4, and CXCL12 mRNA expression compared to control cells [30]. Similarly, nimbolide was shown to downregulate the expression of MMP-9, ICAM-1, and CXCR4 in colorectal cancer xenografts in a nude mouse model [13].

Vascular endothelial growth factor (VEGF) is a pro-angiogenic factor that can be activated by hypoxia-inducible factor-1a (HIF-1a) [74, 75]. Phosphorylated epidermal growth factor receptor (pEGFR) isalso associated with increased angiogenesis in breast cancer [76]. Nimbolide down-regulated expression of both pEGFR and VEGF receptors in breast cancer cells. Moreover, CXCL8 or interleukin-8, a pro-angiogenic chemokine, can stimulate both endothelial cell proliferation and migration through CXCR2 as well as enhance VEGF production [73, 77]. Evidently, nimbolide also reduced expression of CXCL8 and CXCR2 in breast cancers, and thus abrogated angiogenesis [30].

### Chemoprevention

Nimbolide has potential to prevent pro-carcinogen activation and oxidative DNA damage by inhibiting phase I carcinogen activation enzymes (CYP1A1, CYP1B1) and simultaneously induce phase II carcinogen detoxification enzymes (glutathione-S-transferase (GST) and quinonereductase (QR) [7, 13, 62, 78]. Besides modulation of xenobiotics-metabolizing enzymes, nimbolide also upregulates cellular antioxidant content, which aids in decreasing carcinogen-induced oxidative DNA damage, a potentially critical event in neoplastic transformation. In another *in vivo* study withnimbolide in the DMBA-painted buccal pouch of hamster, nimbolidetreatment increased the levels of various antioxidant enzymes (glutathione peroxidase, gamma-glutamyltranspeptidase, superoxide dismutase, and catalase) compared to control group [7].

## IMPORTANCE OF PK STUDIES

Although accumulating evidence from *in vitro* and *in vivo*studes indicated that nimbolide exerts multiple pharmacological effects (antioxidant and anticancer effects), clinical trials to determine the effectiveness of nimbolideas an anticancer drughave not been conducted. The main reason for this is most likely due to the lack of preclinical PK parameters (ADME) of nimbolide. Most natural drug candidates fail to advance in clinical studies due to poor pharmacokinetic properties. Waring et al. (2015) reported that pharmacokinetics and bioavailability remained the third most common cause of attrition, accounting for 16% of failuresof compounds in Phase I trials, among a set of 605 terminated drug candidates developed by 4 major pharmaceutical companies from 2000 to 2010 [79]. For instance, resveratrol, a promising antiaging and anticancer drug candidatederived from grapes, has poor bioavailability despite having favourable *in vitro* data, thus limiting its use in clinical studies [80].

Oral bioavailability is a key factor to ensure an effective drug concentration is physiologically achievable. PK studies can evaluatethe bioavailability of a drug, which is vital for optimizing the administration route and schedule to achieve therapeutic efficacy [81]. In an *in vivo* study of nimbolidein colorectal cancer, plasma levels of 222 and 409ng/mL were detected in colorectal cancer xenografted mice 2h after treatment with nimbolide at 5 and 20 mg/kg *i.p.*, respectively. Nimbolide levels of 345 and 868 ng/g of tumor tissue were obtained from the mice treated with nimbolide at 5 and 20 mg/kg *i.p.*, respectively. The concentrations of plasma and tumour tissue samples were analyzed using high performance liquid chromatography (HPLC) [13]. However, this HPLC method for determination of nimbolide in mouse plasma and tissue has not been fully validated according to the US Food Drug Adminsitration (FDA) guidelines [82].

Distribution of nimbolide within solid tumours and normal tissues is also an important parameter that requires investigation. During the *in vivo* study of nimbolide in glioblastomamultiforme, where brain tumour cells were xenografted either by injection at the flank or into the cerebral cortex, the tumour volumes of nimbolide treatment group were about 40% smaller than those of DMSO control group [12]. The favourable PD results indicated that nimbolide might be able to cross blood-brain barrier (BBB) and the concentration could be sufficient for nimbolide to suppress intracranial tumour cell proliferation. Therefore, *in vivo* PK studies focused on BBB permeability should be carried out to assess blood-brain cerebrospinal fluid and blood-brain extracellular fluid drug concentration relationships, in relation to variation in drug doses and plasma drug levels [83].

More crucially, metabolite evaluation is essential for nimbolide because many natural products are pro-drugs that must undergo metabolic conversion either by the intestinal microflora or mammalian phase I and/or II metabolism before becoming an active metabolite [84]. For instance, Romidepsin (Istodax), isolated fromthe fermentation extract of the rod-shaped bacterium *C. violaceum*, is a prodrug that is converted in cells to its active form by the reduction of the disulfide bond by glutathione. FDA has approved Romidepsin in 2009 to treat cutaneous T-cell lymphoma [85]. It was reported that nimbolide is a CYP1A1 and CYP1A2 inhibitor as well as a phase II enzymes inducer [7], but more studies need to be carried out to determine whether nimbolide has any active metabolite.

To sum up, the bioavailability evaluation of nimbolidebecomesmore critical ifnimbolideis chosen to be developedas a commercial drug for cancer prevention, in which oral administration is required. PK studies performed with robust and sensitive bio-analytical methods allow us to identify ADME constraints and better understand the bioavailability and other important PK parameters of nimbolide, such as the C_max_ (maximum concentration), T_max_ (time to reach maximum concentration), t_1/2_ (elimination half-life), clearance, volume of distribution, and dose linearity/proportionality [86]. So far, there are no systematic studies that have been done to investigate these PK properties of nimbolide. Therefore, the following aspects are urgently needed in getting nimbolide into the clinic. These include i) developing and validating a highly sensitive LC-MS/MS method for determination of nimbolid econcentrations in plasma and tissue samples; ii) performing preclinical PK studies in mice (rodent model), and monkeys (non-rodent model) to define optimized dosage, formulation, and treatment schedule. Based on these preclinical PK parameters, a well-designed phase I clinical trial can be conducted to determine the maximum tolerated dose of nimbolide and response in cancer patients.

## CURRENT STATUS ON TOXICITY STUDIES

While PK is a vital part of drug development, preclinical safety/toxicity testing is very important for the development of new drugs;especially in anticancer drugs, since efficacy does not guarantee non-toxicity. During the period from 2000 to 2010, out of 808 drug candidates developed by 4 major pharmaceutical companies, 356 compounds (44%) failed to progress into clinical studies due to toxicity. Preclinical toxicity was the highest cause of attrition accounting for 59% of the failure at the preclinical phase [79]. Hence, the high toxicity-based attrition further highlights the importance of toxicological studies in drug development. In this section, acute toxicity, mutagenicity, and spermicidal effects of nimbolide will be discussed.

The toxicity of nimbolide in human has not been studied. However, nimbolide exhibited acute toxicity in mice, hamster and rats. Nimbolide was more toxic to mice when given *i.p.*and *i.v.* at same doses, compared to rats and hamsters. Moreover, in adult male mice, the median lethal dose (LD_50_) of *i.p.* route was 225mg/kg, while LD_50_of *i.v.* was 24mg/kg body weight, 10 times more toxic than *i.p.*route. The higher toxicity for *i.v.*mightbe due to higher peak concentration(C_max_) compared to *i.p*., which has an absorption phase. The toxicity of nimbolide in mice is markedly reduced when administered intragastrically/orally (*i.g.*), subcutaneously (*s.c*) and intramuscularly (*i.m.*), with LD_50_ exceeds 600mg/kg body weight [17]. All the above-mentioned toxicological results need to be interpreted based on PK properties of nimbolide, which are currently absent. Hence, a systematic PK study is warranted to determine PK parameters of nimbolide and its metabolite profile.

Furthermore, the spermicidal effect of nimbolide in animals is still inconclusive. In an *in vitro* study, nimbolide had no significant effect on the viability of rabbit sperm [10]. Upon exposure to nimbolide at 50 μM for 60 min, there was only 20-30% reduction in mobility and viability of the rabbit sperm. However, in another *in vivo* study, nimbolide, when administered subcutaneously at dose 0.5-1.5 mg/kg to Wistar strain male albino rats, was shown to reduce sperm functional parameters and increase abnormal sperm countin a dose-dependent manner [87]. This result was supported by the *in vitro* study carried out by the same research group. In this study, 0.5-2 μM of nimbolide was able to deplete the antioxidant defense system in sperm of Wistar strain male albino rats by decreasing activity of antioxidant enzymes (SOD, catalase, GR, GPx); hence increasing the level of production of ROS and inducing oxidative stress in epididymal sperm of rats [88]. With respect to mutagenicity, nimbolide possessed no mutagenic effectusing six tester strains of *Salmonella typhimurium* [4, 89].

Although toxicity was significantly reduced when nimbolide was administered *i.g., s.c.* and *i.m.*, the systemic exposure with repeated doses of nimbolide (repeated-dose toxicity studies) should be thoroughly investigated to identify the no observed adverse effect level (NOAEL) in the most appropriate animal species. According to FDA, both a rodent (rat or mouse) and a non-rodent (dog or monkey) general toxicity study of at least 14 days should be conducted [90]. The preclinical data including dose-response relationship, pharmacokinetics and toxicological profile are important for determination of the starting dose in first-in-human studies [90].

## CONCLUSION AND FUTURE PROSPECTS

Nimbolide has been used in traditional Indian medicine to treat various diseases such as infections, gastric ulcers, and cancer. Accumulating evidence from *in vitro* and *in vivo* studies supports the anticancer and chemopreventive properties of nimbolide in many cancer types including WM, colon, and brain cancer. Multiple mechanisms of action for nimbolide's anticancer and chemopreventive effects have been proposed based on its molecular targets identified in several types of cancer. The main anticancer mechanisms of action ofnimbolide include anti-proliferation, induction of apoptosis, inhibition of angiogenesis and migration, as well as suppression of tumourigenesis via modulation of carcinogen metabolizing enzymes. Compared to the great number of *in vitro* studies that have been conducted, more efforts should be spent on performing *in vivo* studies to verify the *in vitro* findings. In addition, there is a contradiction between *in vitro* concentration and *in vivo* dose in certain types of cancer. Therefore systematic PK evaluationsare urgently required to interpret the inconsistency between *in vitro* and *in vivo* results. To the best of our knowledge, a sensitive bio-analytical method to determine the effective plasma and intracellular concentrations of nimbolide is currently lacking, which is the main obstacle to understanding nimbolide's biologically relevant concentrations in animal models. Furthermore, comprehensive toxicological studies should be conducted to support the safety of nimbolide in animal models including the non-rodent species. Taken together, nimbolide has demonstrated anticancer and chemopreventive effects on many different types of cancer in preclinical studies. Well-designed PK studies and long-term safety/toxicity investigations can greatly accelerate its development as a potential anticancer drug candidate in early phase clinical trials.

## Abbreviations

*A. indica*: *Azadirachtaindica*
ADME: absorption, distribution, metabolism and elimination
Akt: AKT8 virus oncogene cellular homolog
AUC: area under the curve
Bax: Bcl-2-associated X protein
Bcl2: B-cell lymphoma 2
Bcl-xL: B-cell lymphoma-extra-large
CDKs: Cyclin dependent kinases
C_max_: maximum concentration
c-Myc: Avian myelocytomatosis virus oncogene cellular
CYP: cytochrome P450
DMBA: 7,12-Dimethylbenzanthracene
EGFR: Epidermal growth factor receptor
ERK: Extracellular-signal-regulated kinase
GSK-3: Glycogen synthase kinase 3
HIF-1a: hypoxia-inducable factor 1a
ICAM: Intercellular adhesion molecule
IGF-1: Insulin-like growth factor 1
IκB: Inhibitor of kappa B
IKK: IκB kinase
IL-6: Interleukin 6
JAK: Janus kinase
JNK: c-jun N-terminal kinase
KEAP: Kelch-like erythroid-cell- derived protein with CNC homology (ECH)-associated protein
LD_50_: median lethal dose
LEF: Lymphoid enhancer-binding factor
MAPKs: Mitogen-activated protein kinases
MMPs: Matrix metalloproteinases
MOMP: mitochondrial outer membrane potential
NF-κB: Nuclear factor kappa B
NL: Nimbolide
Nrf-2: Nuclear factor (erythroid-derived 2)-like 2
PD: pharmacodynamics
PI3K: Phosphoinositide 3-kinase
PK: pharmacokinetics
PMA: phorbol 12-myristate 13-acetate
ROS: Reactive oxygen species
Smac/DIABLO: second mitochondria-derived activator of caspases
TIMP: tissue inhibitor of metalloproteinase
TNF: tumor necrosis factor
*S. aureus*: *Staphylococcus aureus*
*S. coagulase*: *Staphylococcus coagulase*
STAT: Signal transducer and activator of transcription
t_1/2_: elimination half-life
TEF: Transcription enhancer factor
T_max_: time to maximum concentration
TRAIL: TNF-related apoptosis-inducing ligand
uPA: urokinase plasminogen activator
VEGF: Vascular endothelial growth factor
WM: Waldenstrommacroglobulinemia
XIAP: X-linked inhibitor of apoptosis protein.
